# Age Effects and Temporal Trends in HPV-Related and HPV-Unrelated Oral Cancer in the United States: A Multistage Carcinogenesis Modeling Analysis

**DOI:** 10.1371/journal.pone.0151098

**Published:** 2016-03-10

**Authors:** Andrew F. Brouwer, Marisa C. Eisenberg, Rafael Meza

**Affiliations:** Department of Epidemiology, University of Michigan, Ann Arbor, MI, United States of America; University of California Irvine, UNITED STATES

## Abstract

Differences in prognosis in HPV-positive and HPV-negative oral (oropharyngeal and oral cavity) squamous cell carcinomas (OSCCs) and increasing incidence of HPV-related cancers have spurred interest in demographic and temporal trends in OSCC incidence. We leverage multistage clonal expansion (MSCE) models coupled with age—period—cohort (APC) epidemiological models to analyze OSCC data in the SEER cancer registry (1973–2012). MSCE models are based on the initiation—promotion—malignant conversion paradigm in carcinogenesis and allow for interpretation of trends in terms of biological mechanisms. APC models seek to differentiate between the temporal effects of age, period, and birth cohort on cancer risk. Previous studies have looked at the effect of period and cohort on tumor initiation, and we extend this to compare model fits of period and cohort effects on each of tumor initiation, promotion, and malignant conversion rates. HPV-related, HPV-unrelated except oral tongue, and HPV-unrelated oral tongue sites are best described by placing period and cohort effects on the initiation rate. HPV-related and non-oral-tongue HPV-unrelated cancers have similar promotion rates, suggesting similar tumorigenesis dynamics once initiated. Estimates of promotion rates at oral tongue sites are lower, corresponding to a longer sojourn time; this finding is consistent with the hypothesis of an etiology distinct from HPV or alcohol and tobacco use. Finally, for the three subsite groups, men have higher initiation rates than women of the same race, and black people have higher promotion than white people of the same sex. These differences explain part of the racial and sex differences in OSCC incidence.

## Introduction

In 2013, the National Cancer Institute published its Annual Report to the Nation on the Status of Cancer, 1975–2009, highlighting the trends in the burden of human papillomavirus (HPV) associated cancers in the United States. Although total cancer incidence has recently declined, incidence of HPV-positive oropharyngeal (OP) cancers have increased proportionally [1, 2], so much so as to be called an epidemic by some [3]. There appear to be two major etiologies of head and neck squamous cell carcinomas (HNSCC), one with alcohol and tobacco use as predominant etiologic factors [4], and one related to HPV infection and subsequent HPV genome integration, each with its own prognosis, risk-factor profiles, and genetic markers [5]. HPV-positive cancers appear to be limited to certain subsites of the head and neck, particularly in the oropharyngeal region, and, on the basis of molecular and epidemiologic data, head and neck subsites have been designated as HPV-related or HPV-unrelated [6–8]. Not all cancers at HPV-related sites are HPV-positive, but the classification is helpful in the absence of information about tumor HPV-status in cancer registries.

Analysis of HPV-related oral (oropharyngeal and oral cavity) squamous cell carcinomas (OSCC) incidence in the Surveillance, Epidemiology, and End Results (SEER) cancer registries, have identified gender disparities but diminishing racial differences in the United States [7, 8]. Overall OSCC incidence rates for men are two to four times that of women across all races, though this varies slightly for the different cancer subsite groups [8]. Racial differences between rates of OSCC in white and black women have largely disappeared. Although rates for black men have historically been higher than for white men, declining rates among black men have been met by a recent increase in incidence for white men [7]. These results, however, only address overall temporal trends and neither distinguish between age, period, and birth cohort trends nor make implications about the underlying biological and epidemiological causes. Multistage clonal expansion (MSCE) models, a class of Markov models, capture the initiation—promotion—progression hypothesis of tumorigenesis, in which normal cells undergo a genetic transformation that leads to clonal expansion, followed by transformations that lead to malignancy [9–12]. Using models that account for the natural history of cancers is important because the effects of carcinogens acting as initiators or promoters result in different temporal trends in the the age-specific incidence of cancer, which can be inferred from population level data [12, 13]. MSCE models have been shown to capture temporal patterns of cancer risk and provide insight into the underlying mechanisms leading to population-level cancer incidence patterns [11, 12, 14, 15]. Here we use MSCE models adjusted for temporal trends to analyze the incidence of HPV-related and -unrelated OSCC. We demonstrate that MSCE models with both period and birth cohort temporal effects can better identify temporal trends and place them in the context of putative underlying cancer mechanisms.

## Methods

### Data

We consider a subset of head and neck cancers reported to the Surveillance, Epidemiology, and End Results (SEER) cancer registries. We use the International Classification of Diseases (ICD) codes, as in Chaturvedi et al. [6] and Brown et al. [7, 8] to group sites into HPV-related, HPV-unrelated except for oral tongue, and HPV-unrelated oral tongue. A full list of the codes and sites is provided in the supplementary information (S1 File). For brevity, we will henceforth denote these subgroups as HPV-related, HPV-unrelated, and oral tongue, respectively.

We use SEER 9 data 1973–1991, SEER 13 data 1992–1999, and SEER 18 data 2000–2012, in order to leverage the increased sample size in later years. We refer to this data set as SEER Max. Concerns that can arise when combining these data sets—relating to changing racial composition, urban/rural divides, and the geographical distribution of new SEER registries— are minimized in this study by performing separate analyses for white men, black men, white women, and black women. Only white and black races are considered in this analysis, and we do not stratify by Hispanic/non-Hispanic ethnicity; SEER reports incidence rates by ethnic origin only for all races combined, white, and non-white. We consider only ages 0–84, as all cases for ages 85 and over are aggregated in the database. Table 1 gives the total number of cases for ages 0–84 for each group between 1973 and 2012. Case and population data are provided as supplmentary files (S2–S17 Files).

**Table 1:** Number of cases of oral (oropharyngeal and oral cavity) squamous cell carcinoma among ages 0–84 between 1973–2012 by race and cancer subsite group.

Demographic	HPV-related	HPV-unrelated	Oral tongue
White men	35,349	28,687	1,1798
Black men	4,762	4,859	1,021
White women	9,599	14,101	7,607
Black women	1,374	1,616	513

### Two-stage clonal expansion models

**Fig 1:**
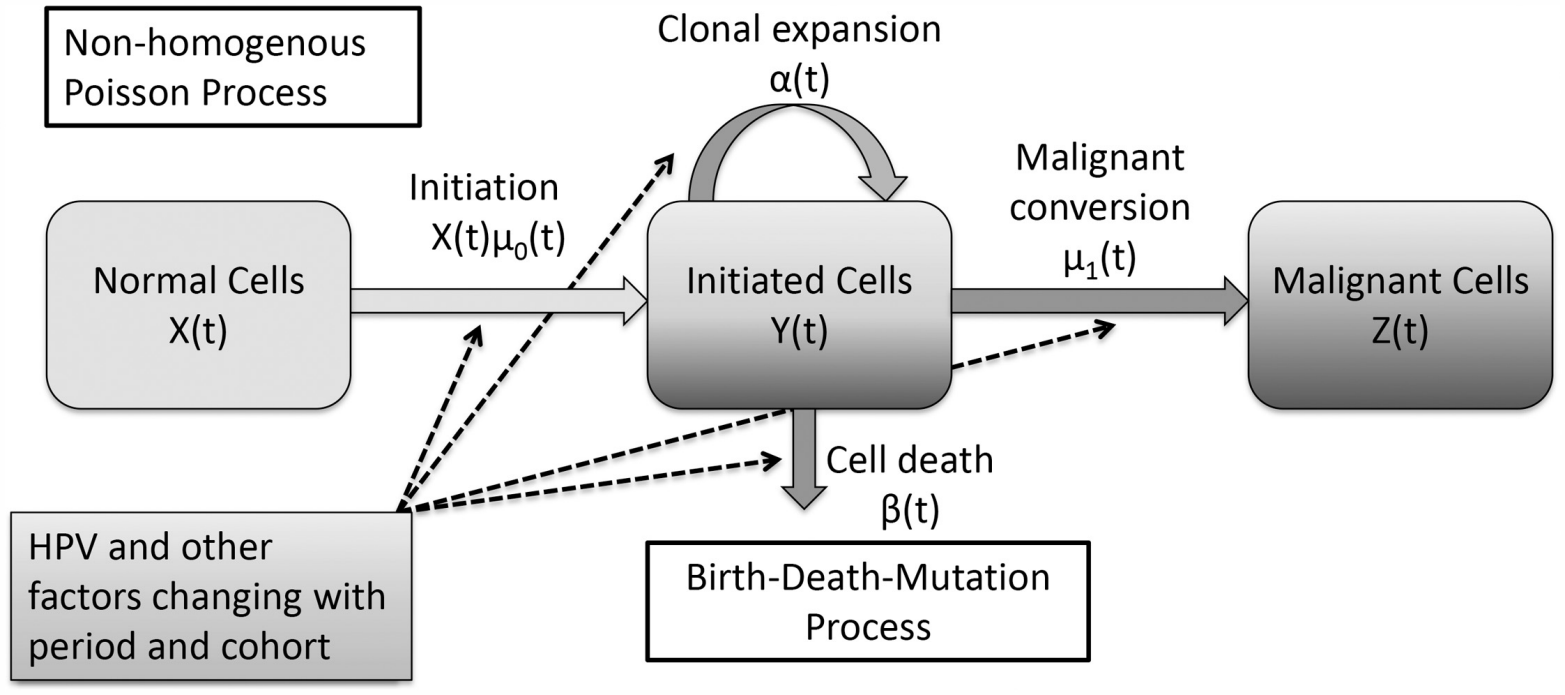
Schematic of the two-stage clonal expansion model with period and cohort dependencies.

The two-stage clonal expansion (TSCE) model was developed by Moolgavkar, Venzon, and Knudson [9, 10] to capture the initiation—promotion—progression paradigm. Moolgavkar, Venzon, and Knudson described initiation through a non-homogenous Poisson process and clonal expansion and malignant conversion through a birth—death—mutation process, the details of which are described at length elsewhere [9, 10, 12, 16]. There are five, possibly age-dependent, parameters: initial number of uninitiated cells *X*(0), initial mutation rate *μ*_0_(*t*), growth rate *α*(*t*) and death rate *β*(*t*) of initiated cells, and malignant mutation rate *μ*_1_(*t*). In Fig 1, we present a schematic of the model, which includes the possible time-dependent effects of HPV or other factors on the parameters.

If the parameters *α*, *β*, *μ*_0_, and *μ*_1_ are not age-dependent, the closed form solutions for the survival and hazard are

$$\begin{array}{c} S(t)={\left(\frac{q-p}{qe^{-pt}-pe^{-qt}}\right)}^{r}, \end{array}$$ (1)

$$\begin{array}{c} h(t)=\frac{rpq(e^{-qt}-e^{-pt})}{qe^{-pt}-pe^{-qt}}, \end{array}$$ (2)

where

$$\begin{array}{c} r=\frac{\mu_{0}X\left(0\right)}{\alpha }, \end{array}$$ (3)

$$\begin{array}{c} p,q=\frac{1}{2}\left(-\left(\alpha -\beta -\mu_{1}\right)\mp \sqrt{{\left(\alpha -\beta -\mu_{1}\right)}^{2}+4\alpha \mu_{1}}\right). \end{array}$$ (4)

The model parameters themselves are not all identifiable given age-specific incidence data, but *r*, *p*, and *q* constitute identifiable combinations. Note that −(*p* + *q*) is the net clonal cell proliferation *α* − *β* − *μ*_1_, and *pq* = −*αμ*_1_. Further, $q\approx \mu_{1}/\left(1-\frac{\beta }{\alpha }\right)$ and *p* ≈ − (*α* − *β*) [17]. Hence, we identify (multiplicative) effects on *r* with effects on initiation, effects on *p* with effects on promotion, and effects on *q* with effects on malignant conversion.

Under the TSCE model, the sojourn time *T*_*s*_ of a tumor, the time between the time of tumor onset (first premalignant mutation) and the time of clinical detection can be approximated by

$$\begin{array}{c} T_{s}\approx -\frac{\ln(q/(-p)}{-p}\approx -\frac{\ln\left(\alpha \mu_{1}/{\left(\alpha -\beta \right)}^{2}\right)}{\alpha -\beta } \end{array}$$ (5)

(as long as *μ*_1_ ≪ 1 and *μ*_1_ < *p*^2^/*α*) [12, 15, 18].

In the general case of age-dependent parameters, that is *α*, *β*, *μ*_0_, and *μ*_1_ are arbitrary functions of *t*, numerical solutions can be found [19, 20], although we will not consider that case in this investigation.

This model formulation may be extended to three stages and other more complex models and has been applied successfully to a variety of cancer types [11, 12, 14, 19, 21–27]. Models with more pre-initiation stages—more “hits” before initiation—have different asymptotic behavior than the two-stage model, and, although they have been shown to be appropriate for cancers like pancreatic and colorectal [12], the two-stage model fits the oral cancer data better in most cases. A comparison of model fits for the two- and three-stage models is provided in the supplement (S1 File).

### Age—period—cohort models

Age—period—cohort (APC) models are a class of epidemiological models used to disentangle effects of age, period (factors affecting all people alive at a given time), and birth cohort (factors affecting all people born in a given time period) given prevalence (e.g. HPV prevalence) or incidence (e.g. incidence of oral cancer). The traditional model posits that incidence rates λ are described by a multiplicative model with age (*A*), period (*P*), and birth cohort (*C*) [28–31]. This is usually treated in the logarithmic form, in which we fit the model

$$\begin{array}{c} \log\lambda =\beta_{0}+\beta_{A}\left(A\right)+\beta_{P}\left(P\right)+\beta_{C}\left(C\right), \end{array}$$ (6)

where *β*_0_ is a constant and *β*_*A*_, *β*_*P*_, and *β*_*C*_ are some functions to be determined, often constrained to be discrete functions or splines.

One drawback of full APC models is their inherent unidentifiability: *P* = *A* + *C*. To resolve the unidentifiability, one may consider only two effect models, typically age—period or age—cohort, or constrain the age effects to have a given shape, such as the hazard function of a MSCE model, as we do in this analysis [11, 12, 15, 29].

Given a set of observed cases {*x*_*i*_} with corresponding population-at-risk sizes {*n*_*i*_}, we derive a likelihood for the APC model in the following way. We assume that observed incident cases *x*_*i*_ for a given population all of age *A*_*i*_ at time *P*_*i*_ from birth cohort *C*_*i*_ = *P*_*i*_ − *A*_*i*_ are Poisson distributed with mean *μ*_*i*_ = *n*_*i*_ ⋅ λ(*A*_*i*_, *P*_*i*_, *C*_*i*_), where λ is the incident rate function dependent on parameter *β*_0_ and functions *β*_*A*_, *β*_*P*_, and *β*_*C*_. Observations are assumed to be independent, and thus the likelihood for the whole data set of observations {*x*_*i*_} is given by

$$\begin{array}{c} L(\beta_{0},\beta_{A},\beta_{P},\beta_{C})=\prod_{i}\frac{e^{-\mu_{i}}\mu_{i}^{x_{i}}}{x_{i}!}. \end{array}$$ (7)

In this context of cancer diagnosis, interpretation of age and cohort effects is straightforward. In contrast, the period effect on its own represents a time-of-diagnosis effect, which may not accurately describe the historical effect of period on cancer. However, in concert with cohort effects, it becomes a surrogate measure of a person’s cumulative period effects. We follow this approach rather than explicitly modeling a historic period trend—i.e. period effects covering all calendar years since birth—as this would introduce an additional identifiability problem.

### APC—TSCE hybrid models

In a general APC model, the age effects are not constrained, but if we are working within the TSCE framework, we can restrict the age effects to have the shape of the TSCE hazard:

$$\begin{array}{c} \log\lambda =\beta_{0}+\log\left[h(t,r,p,q)\right]+\beta_{P}\left(P\right)+\beta_{C}\left(C\right). \end{array}$$ (8)

This added constraint theoretically resolves the non-identifiability problem in the full APC model [15, 29]. In the case of constant parameters, the multiplicative assumption of the model translates to an assumption that the period and cohort effects are on the rate of initiation *μ*_0_ since *r* = *μ*_0_ *X*(0)/*α* and *X*(0) and *α* are considered fixed:

$$\begin{array}{c} \lambda =-\left[f(P,C)\cdot r\right]\left(\frac{pq(e^{-qt}-e^{-pt})}{qe^{-pt}-pe^{-qt}}\right). \end{array}$$ (9)

However, depending on the mechanism of carcinogenesis for a given cancer and the nature of the risk factors captured by the temporal trends, it is possible that the effects on promotion or malignant conversion rates rather than initiation rates are more realistic. Thus, by considering slightly different models with period or cohort effects acting on the promotion or malignant conversion parameters, one can investigate the impact of period and cohort on different stages of carcinogenesis. In this analysis we consider models of the form

$$\begin{array}{c} \lambda =h(t,r(P,C),p(P,C),q(P,C)). \end{array}$$ (10)

Here, **r**(*P*, *C*) = *r* · *θ*_*P*_(*P*) · *θ*_*C*_(*C*) where *θ*_*P*_ and *θ*_*C*_ are natural splines, *r* is the value of **r** at the reference period and cohort, and **p** and **q** are defined similarly.

### Parameter estimation

The negative log-likelihood (NLL) for observed cases {*x*_*i*_} with corresponding population-at-risk sizes {*n*_*i*_} under these models is given by

$$\begin{array}{c} \text{NLL}\left(r,p,q,{\left\{\theta_{P},\theta_{C}\right\}}_{r},{\left\{\theta_{P},\theta_{C}\right\}}_{p},{\left\{\theta_{P},\theta_{C}\right\}}_{q}\right)=-\sum_{i}\left(-\mu_{i}+x_{i}\log\mu_{i}-\log x_{i}!\right), \end{array}$$ (11)

where *μ*_*i*_ = *n*_*i*_ ⋅ λ(*r*, *p*, *q*, *P*_*i*_, *C*_*i*_) and {*θ*_*P*_, *θ*_*C*_} are the parameters of the natural spline functions. The negative log-likelihood, under the assumptions of each model, was minimized using a Davidon-Fletcher-Powell optimization algorithm in R (v. 3.1) [32], and 95% Wald confidence intervals for the biological parameters were calculated from the Hessian. We use the Akaike Information Criterion (AIC) as a measure of model fit. We use the formulation AIC = 2(NLL + *k*) where *k* is the number of parameters. Hence, a more negative AIC represents a better fit, and one point gain in the negative log-likelihood is equivalent to reducing model complexity by one parameter.

Uncertainty quantification was investigated using Markov chain Monte Carlo (MCMC) methods; in particular, a covariance matrix for the parameters was estimated and used to create confidence intervals for the hazards and period and cohort effects. Results and additional details are found in the supplement (S1 File).

## Results

### Age-adjusted incidence

**Fig 2:**
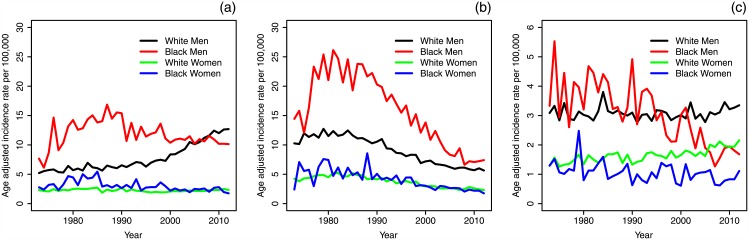
Age-adjusted incidence rates of oral squamous cell carcinoma among ages 30–84 by cancer subsite group: (a) HPV-related, (b) HPV-unrelated, and (c) oral tongue. Please note the change in axes scale for the oral tongue cancer.

Fig 2 shows the age-adjusted incidence rates of oral squamous cell carcinomas (OSCCs) reported in SEER Max (1973–2012) by subsite group; rates are adjusted to the U.S. population in the year 2000. For HPV-related sites, incidence rates in white and black women show little to no trend. There appears to be a slight downward trend for black men, but a clear upward trend is seen for white men. For HPV-unrelated sites, all four groups peak in the early 1980s and trend down afterward. For oral tongue sites, incidence for white men has remained relatively constant, black men and black women have trended down (slightly for women and more strongly for men), and white women have trended slightly up.

### Incidence by period and cohort

**Fig 3:**
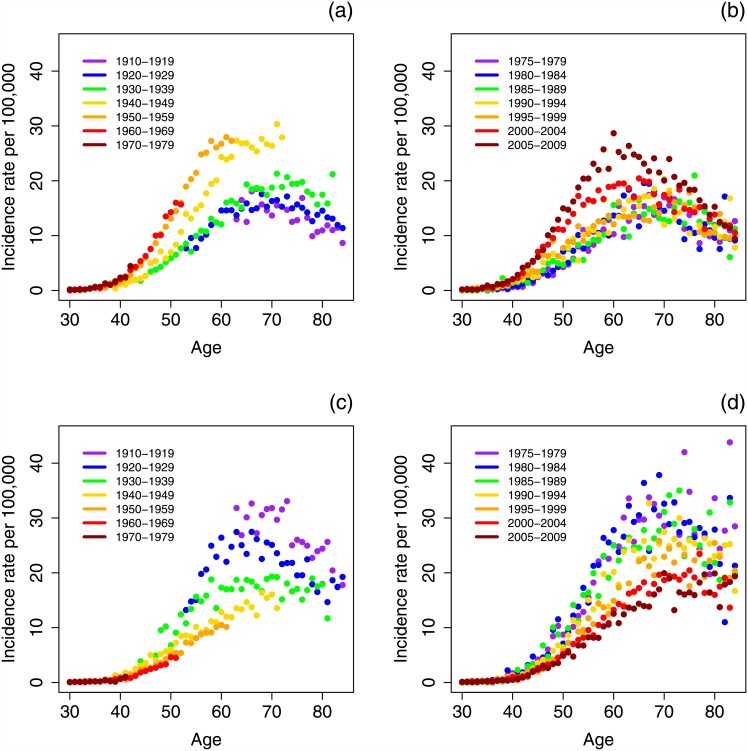
Incidence rates of oral squamous cell carcinoma among white males for HPV-related, by cohort (a) and period (b), and HPV-unrelated subsite groups, by cohort (c) and period (d).

Fig 3 shows OSCC incidence rates for selected calendar years and birth-cohorts for white males. In Fig 3a, it appears that incidence of HPV-related OSCCs increased dramatically for the birth cohorts between 1940 and 1970. When age-specific incidence is stratified by period, we see an increasing trend after the early 1990s (Fig 3b). For the HPV-unrelated OSCCs, we see a decrease in incidence with each birth cohort decade as well as by period (Fig 3c and 3d). Yearly variation in incidence make interpretation of the other race—cancer-site pairs difficult, and, thus, the analogous figures are left to the supplement (S1 File).

### APC—TSCE model results

**Fig 4:**
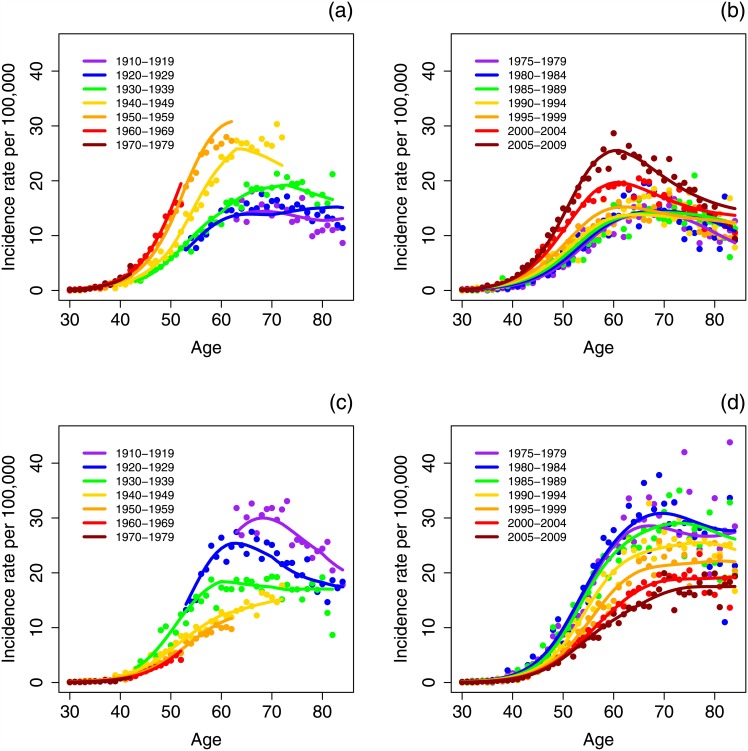
Fits of the two-stage clonal expansion model with period and cohort effects on initiation to incidence rates of oral squamous cell carcinoma among white males for HPV-related subsites, by cohort (a) and period (b), and HPV-unrelated subsites, by cohort (c) and period (d). The dots are SEER data, and lines are model fits.

We constrained the age effects to the form of the TSCE hazard, and considered only ages 30–84. We investigated period and cohort effects on our proxies for tumor initiation *r*, promotion *p*, and malignant conversion *q*. Further, to force the cohort and period effects into a more realistic form, we used natural splines with eight degrees of freedom for cohort effects and five for period, corresponding to approximately one degree of freedom for twelve and eight years respectively. We chose one model for all demographics in each of the three cancer subsite groups. We determined that period and cohort effects on initiation (*r*) gave the best model fits for all three subsite groups as this model gave the lowest AIC for most demographics in each category. A table of AIC for the considered models is shown in the supplement (S1 File). Fits of the selected APC—TSCE model to the data for HPV-related and HPV-unrelated cancers in white men are plotted in Fig 4 as a companion to Fig 3. Model fits to the other demographics and subsite groups are included in the supplement (S1 File). For comparison to this APC—TSCE model, we fitted the standard, unconstrained APC model as well; these results are also shown in the supplement (S1 File).

**Fig 5:**
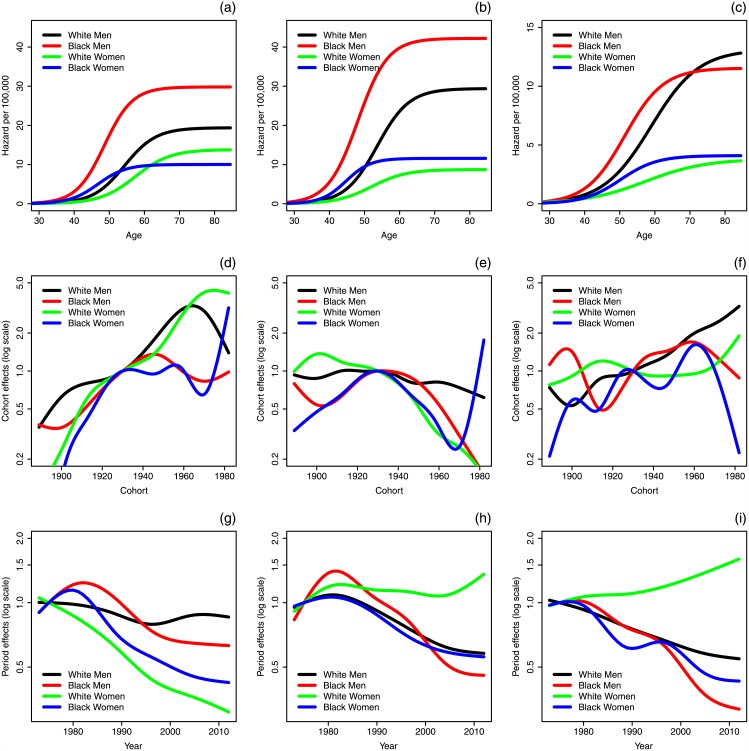
Hazard, cohort effects, and period effects for the cohort-and-period-effects-on-*r* APC—TSCE models of oral squamous cell carcinoma by race and cancer subsite group. (a) HPV-related hazards. (b) HPV-unrelated hazards. (c) Oral tongue hazards. (d) HPV-related cohort effects. (e) HPV-unrelated cohort effects. (f) Oral tongue cohort effects. (g) HPV-related period effects. (h) HPV-unrelated period effects. (i) Oral tongue period effects.

In Fig 5a–5c, we present the estimated model hazard (age-specific cancer incidence function) for HPV-related, HPV-unrelated, and oral tongue OSCCs for each of the four considered groups under the model that considers cohort and period effects on *r*. In terms of the model parameters, the initiation parameter *r* determines the hazard’s asymptote level, whereas the promotion parameter *p* determines the rate of increase in the hazard in the middle adult ages. Thus a higher asymptote is suggestive of higher initiation rates in a given demographic group, and an earlier and more rapid increase in the hazard in middle ages suggests higher promotion rates. For all three OSCC subsite groups, the hazard begins to increase earlier for black men than white men and earlier for black women than white women, and the asymptotes for white and black men are higher than for white and black women. This then suggests higher promotion rates in blacks, and higher initiation rates in men. The shape of the hazard for the oral tongue sites is qualitatively different from that of the other two.

Cohort effects and period effects are plotted for this model in Fig 5d–5i. The cohort and period effects are plotted on a log-scale to emphasize that, for example, an effect of 0.5 and an effect of 2 are equally different from the reference. To interpret the period and cohort effects, note that the product of the two effects gives the modeled relative initiation rate, and hence the incidence, compared to that of the members of the reference group, here the 1930 birth cohort in 1975.

We observe that, for HPV-related OSCCs, there is a five-fold increase in relative cohort effect for white women between 1900 and 1930 followed by another five-fold increase between 1930 and 1980, a dramatic change overall. Further, the overall cohort trends for the oral tongue subsites are somewhat similar to those of HPV-related subsites, while those of the HPV-unrelated are different from the other two. Finally, all three cancer subsite groups show decreasing period trends for most demographics, though the period effects for HPV-unrelated and oral tongue OSCC incidence for white women do not follow this pattern.

The estimates of the biological parameters for initiation *r*, promotion *p*, and malignant conversion *q* are presented in Table 2. The values of the biological parameters display some clear patterns. For all three cancer subsite groups, men have a larger initiation *r* than women, regardless of race, and black men and women have a larger promotion *p* than their white counterparts. Further, the promotion *p* is very similar for the HPV-related and HPV-unrelated subsites. As discussed above, we can see the effect of the estimated biological parameters reflected in the plot of the hazards (Fig 5a–5c). As mentioned before, a larger initiation *r* generally corresponds to a higher final hazard asymptote (with the exception of black and white women in the HPV-unrelated plot), since the asymptotic value of the hazard *h*(*t*) is *r* ⋅ (−*p*) and the deviance in *p* is relatively small. Further, a larger promotion *p* corresponds to an earlier increase in the hazard. We estimate mean sojourn time *T*_*s*_ from the biological parameters: black men and women have shorter sojourn times, and, generally, the sojourn time is shorter for women than for men. White men and women for HPV-related OSCCs are the one exception.

**Table 2:** Biological parameters for the period-and-cohort-on-*r* APC—TSCE models of oral squamous cell carcinoma by race and cancer subsite group with 95% Wald confidence intervals.

Data	*T*_*s*_	*r*	Low *r*	High *r*	*p*	Low *p*	High *p*	*q*	Low *q*	High *q*
**HPV related**										
White men	54.3	8.72E-4	7.58E-4	1.00E-3	-2.23E-1	-2.29E-1	-2.16E-1	1.25E-6	9.75E-7	1.60E-6
Black men	48.6	1.19E-3	8.89E-4	1.60E-4	-2.50E-1	-2.74E-1	-2.28E-1	1.31E-6	5.90E-7	2.91E-6
White women	57.4	6.91E-4	5.26E-4	9.09E-4	-2.00E-1	2.09E-1	-1.91E-1	2.05E-6	1.32E-6	3.19E-6
Black women	46.7	3.98E-4	2.44E-4	6.48E-4	-2.52E-1	-3.94E-1	-2.09E-1	1.91E-6	3.94E-7	9.24E-6
**HPV unrelated**										
White men	53.6	1.34E-3	1.17E-3	1.53E-3	-2.20E-1	-2.28E-1	-2.12E-1	1.69E-6	1.25E-6	2.30E-6
Black men	48.0	1.80E-3	1.37E-3	2.36E-3	-2.35E-1	-2.59E-1	-2.13E-1	2.93E-6	1.35E-6	6.37E-6
White women	52.0	4.32E-4	3.54E-4	5.28E-4	-2.02E-1	-2.18E-1	-1.87E-1	5.52E-6	3.11E-6	9.79E-6
Black women	44.7	4.02E-4	2.72E-4	5.93E-4	-2.88E-1	-3.48E-1	-2.34E-1	7.36E-7	9.57E-8	5.66E-6
**Oral tongue**										
White men	58.9	8.93E-4	6.52E-4	1.22E-3	-1.47E-1	-1.54E-1	-1.40E-1	2.58E-5	1.59E-5	4.17E-5
Black men	51.4	6.37E-4	2.90E-4	1.40E-3	-1.81E-1	-2.18E-1	-1.49E-1	1.64E-5	5.11E-6	5.25E-5
White women	57.4	3.27E-4	1.98E-4	5.40E-4	-1.16E-1	-1.27E-1	-1.06E-1	1.45E-4	7.58E-5	2.78E-4
Black women	50.2	2.13E-4	8.58E-5	5.30E-4	-1.92E-1	-2.45E-1	-1.49E-1	1.24E-5	2.14E-6	7.21E-5

## Discussion

### Main findings

Trends in incidence of carcinoma at the three groups of subsites of the oropharynx and oral cavity, namely HPV-related sites, HPV-unrelated sites (except for oral tongue), and oral tongue sites, appear to be primarily driven by period and birth cohort effects on the cancer initiation rate rather than the cancer promotion rate or malignant conversion rate. For all three subsite groups, too, men had higher estimated initiation rates than women of the same race, and black men and women had higher estimated promotion rates than white people of the same sex. Cancer at the HPV-related and HPV-unrelated sites had very similar estimated promotion rates, which were different from those of cancers of the oral tongue.

The three subsite groups have largely distinct patterns of period and birth cohort effects on their estimated initiation rates. HPV-related carcinomas, for instance, are found to be strongly cohort driven in this analysis, a result that is consistent with other findings that prevalence of sexually transmitted infections are usually related to cohort factors [33]. In particular, we previously found that HPV prevalence itself to be strongly cohort driven [34]. Use of alcohol and tobacco is also strongly cohort driven, and cohort trends in their use [35, 36] are reasonably consistent with the estimated cohort trends for HPV-unrelated oral cancer. Although the overall patterns between the subsite groups are distinct, there are some similarities. The period effects for all three sites have similar trajectories, with the notable exception of white women, which could be related to their lower incidence. Additionally, the cohort effects for the oral tongue sites are similar to, if less pronounced than, those for the HPV-related sites, for all demographic groups but white women. This finding may suggest the etiology of cancer at the oral tongue sites may also be influenced by changes in sexual behavior.

The separation of cohort and period effects in this analysis reveals some trends that were not apparent from the age-adjusted incidence rates alone. In particular, the age-adjusted rates for HPV-related OSCCs in white women remain nearly constant, but this analysis suggests that this seeming lack of trend belies a combination of increasing cohort and decreasing period trends. A similar effect is seen for the age-adjusted rates of oral tongue cancer for white men. Trends in the age-adjusted incidence may be a result of factors that affect everyone in a given time period or, more subtly, be caused by changes between one birth cohort to the next. One can begin to see the effects of these factors when plotting age-specific incidence stratified by different time periods or birth cohorts, as we do for white men for the HPV-related and HPV-unrelated subsite groups in Fig 3. Trends in period or birth cohort for age-specific incidence can either exaggerate trends in the age-adjusted incidence when the period and birth cohort trends align or be masked when the trends are opposing. However, the trends in period and cohort can sometimes be difficult to see in the data alone, especially for relatively rare diseases that have large variation in incidence, and so the results of the age—period—cohort models are especially valuable.

As we saw in Fig 5, the model hazard begins to increase earlier in life for black men and women for all of the subsite groups, which is reflected in the higher estimated cancer promotion rates for those demographics. Although one might, if looking only at the HPV-related figure, conjecture that higher oral prevalence of HPV among black Americans could be the cause, it seems more likely, taking the analysis of the other two groups into account, that it is a factor of other differences in the two populations (smoking, drinking, or other risk factors and exposures). Further, men of both races have higher hazards than the women of the same race, which is reflected, in part, in higher estimated rates of cancer initiation. Again, although this is consistent with men having higher oral prevalence of HPV than women, the consistency across the subsite groups suggests that this effect might be more likely due to the underlying differences in biology.

Analysis of the estimated biological parameters for the three groups, the promotion parameter *p* in particular, suggests that HPV-related and HPV-unrelated cancers are distinct from the cancer of the oral tongue. Interestingly, the estimated rates of promotion *p* and the sojourn times are very similar between the HPV-related and HPV-unrelated OPSCCs and are quite different from those of oral tongue cancer, which seems to progress more slowly; the mean sojourn time for the oral tongue sites is about 2–5 years longer than the other two. The similarity between the HPV-related and HPV-unrelated promotion parameters and estimated sojourn times suggest that the tumor dynamics are very similar for these sites once the tumor has been initiated, whether by HPV, alcohol and tobacco use, or other cause. This similarity may appear to be in contrast with the known differences in cancer survival between HPV-related and HPV-unrelated cancers [1], though factors other than rate of tumor growth affect survival rates.

### Comparison to other literature

To better understand if the differences between the promotion parameters at the different subsite groups of the oropharynx and oral cavity are significant (HPV-related: −0.20 to −0.25; HPV-unrelated: −0.20 to −0.29; oral tongue: −0.12 to −0.19), we compare with findings from colorectal and esophogeal adenocarcinoma. Estimates of the promotion parameters for colorectal adenocarcinomas in United States (SEER) were −0.14, −0.19, and −0.20 for men for the proximal colon, distal colon, and rectum, and −0.14, −0.18, and −0.18 for women [37]. Estimates of the promotion parameter *p* for esophageal adenocarcinoma (SEER) in white men and women range from −0.16 to −0.20 [22]. Hence, this analysis suggests that not only are we seeing significant differences between the dynamics of oral tongue carcinoma and the other sites, but also among the demographics for each subsite group.

That the promotion parameter for cancer of the oral tongue is significantly different from that of the other two is consistent with the findings of other recent studies [8, 38, 39] that observed that patterns of age- and sex-specific incidence of oral tongue cancer seemed to distinguish it from the other sites. This has led to suggestions that cancer of the oral tongue may have a different etiology from either smoking and drinking [38] or HPV [38, 39], possibly related to bacterial or viral infection or genetic abnormalities [8, 38]. Our analysis of period and cohort effects suggests that changes in oral tongue cancer incidence by birth cohort are somewhat similar to those for HPV, which may suggest that the etiology of oral tongue cancer is also linked to changing sexual mores and practices.

### Strengths and limitations

As with any mathematical model, the modeling framework underlying this analysis is a simplification of the complex biological underpinnings of tumorigenesis and thus neglects a number of relevant factors. Additionally, as with other SEER-based studies, our work is limited by the uncertainty in classification of sites as presumed HPV-related or HPV-unrelated. Similarly, this analysis is limited by the lack of alcohol and tobacco consumption data in SEER, which precludes the possibility of controlling for these important risk factors.

The use of a multistage model rooted in the biology of the system, a model which has been previously developed and validated, offers several advantages in this context. In particular, in addition to assessing trends in the data, we are able to pose hypotheses on the biological implications (i.e. the initiation, promotion, and malignant conversion rates and sojourn times); previous studies have not included biologically motivated carcinogenesis models in their analyses. Additionally, the large sample size afforded by the SEER database allows analysis stratified by both sex and race.

### Implications

This analysis suggests that cancer at HPV-related and HPV-unrelated sites have similar tumor growth dynamics once initiated. More work is needed to investigate these dynamics, as survival rates for HPV-positive and HPV-negative tumors are drastically different. Testing for HPV in oropharyngeal carcinomas should become routine and the results recorded in cancer registries.

Although we cannot explicitly test the relationship between alcohol and tobacco use and oral cancer—SEER does not contain these covariates—the differences in cohort and period trends support the hypothesis that oral tongue cancer may have a different etiology from either HPV or alcohol and tobacco use. Although, there is little evidence as to what this etiology is, our analysis offers some additional information that may be useful to future studies. In particular, the birth cohort trends for oral tongue cancer appear similar to that of cancer of the HPV-related subsites, suggesting trends in sexual behavior may be relevant. That white women have distinctly different period effect trends for HPV-related (decreasing) and oral tongue (increasing) cancer while the three other demographics have similar decreasing trends for both groups may offer additional clues, though it is not clear at this time what those might be.

Further, work is needed to understand why men have higher rates of initiation than women, for both white and black Americans, at all three subsite groups, a phenomenon that may be biologically rooted, and why black men and women have higher promotion rates than their white counterparts, a result of risk factors more likely influenced by socioeconomics and behavior than biology.

Future studies may be able to include a joint analysis of HPV prevalence and incidence of HPV-related oropharyngeal squamous cell carcinomas using extensions of the two-stage carcinogenesis model. Indeed, such models may be able to shed light on the similarities and differences in initiation and growth of HPV-related and HPV-unrelated tumor as well as help quantify the additional risk for oral cancer associated with HPV infection.

## Supporting Information

S1 File Supplemental information. Provides additional details on subsite classification and model selection, fit, and uncertainty quantification.
(PDF)

S2 File Population of black women ages 0 to 84, 1973–2012. (CSV)

S3 File Population of black men ages 0 to 84, 1973–2012. (CSV)

S4 File Population of white women ages 0 to 84, 1973–2012. (CSV)

S5 File Population of white men ages 0 to 84, 1973–2012. (CSV)

S6 File Counts of HPV-related cancer for black women ages 0 to 84, 1973–2012. (CSV)

S7 File Counts of HPV-related cancer for black men ages 0 to 84, 1973–2012. (CSV)

S8 File Counts of HPV-related cancer for white women ages 0 to 84, 1973–2012. (CSV)

S9 File Counts of HPV-related cancer for white men ages 0 to 84, 1973–2012. (CSV)

S10 File Counts of HPV-unrelated cancer for black women ages 0 to 84, 1973–2012. (CSV)

S11 File Counts of HPV-unrelated cancer for black men ages 0 to 84, 1973–2012. (CSV)

S12 File Counts of HPV-unrelated cancer for white women ages 0 to 84, 1973–2012. (CSV)

S13 File Counts of HPV-unrelated cancer for white men ages 0 to 84, 1973–2012. (CSV)

S14 File Counts of oral-tongue cancer for black women ages 0 to 84, 1973–2012. (CSV)

S15 File Counts of oral-tongue cancer for black men ages 0 to 84, 1973–2012. (CSV)

S16 File Counts of oral-tongue cancer for white women ages 0 to 84, 1973–2012. (CSV)

S17 File Counts of oral-tongue cancer for white men ages 0 to 84, 1973–2012. (CSV)

## References

[1] Chaturvedi AK, Engels EA, Pfeiffer RM, Hernandez BY, Xiao W, Kim E, et al Human papillomavirus and rising oropharyngeal cancer incidence in the United States. Journal of Clinical Oncology. 2011;29(32):4294–301. 10.1200/JCO.2011.36.4596 21969503 PMC3221528

[2] Jemal A, Simard EP, Dorell C, Noone AM, Markowitz LE, Kohler B, et al Annual Report to the Nation on the Status of Cancer, 1975–2009, Featuring the Burden and Trends in Human Papillomavirus (HPV)-Associated Cancers and HPV Vaccination Coverage Levels. Journal of the National Cancer Institute. 2013;105(3):175–201. 10.1093/jnci/djs491 23297039 PMC3565628

[3] Marur S, D’Souza G, Westra WH, Forastiere AA. HPV-associated head and neck cancer: a virus-related cancer epidemic. Lancet Oncology. 2010;11(8):781–9. 10.1016/S1470-2045(10)70017-6 20451455 PMC5242182

[4] Sturgis EM, Wei Q, Spitz MR. Descriptive epidemiology and risk factors for head and neck cancer. Seminars in Oncology. 2004;31(6):726–733. 10.1053/j.seminoncol.2004.09.013 15599850

[5] Gillison ML, Alemany L, Snijders PJF, Chaturvedi A, Steinberg BM, Schwartz S, et al Human papillomavirus and diseases of the upper airway: head and neck cancer and respiratory papillomatosis. Vaccine. 2012;30 Suppl 5:F34–54. 10.1016/j.vaccine.2012.05.070 23199965

[6] Chaturvedi AK, Engels EA, Anderson WF, Gillison ML. Incidence trends for human papillomavirus-related and -unrelated oral squamous cell carcinomas in the United States. Journal of Clinical Oncology. 2008;26(4):612–9. 10.1200/JCO.2007.14.1713 18235120

[7] Brown LM, Check DP, Devesa SS. Oropharyngeal cancer incidence trends: diminishing racial disparities. Cancer Causes & Control. 2011;22(5):753–63. 10.1007/s10552-011-9748-1 21380619

[8] Brown LM, Check DP, Devesa SS. Oral cavity and pharynx cancer incidence trends by subsite in the United States: changing gender patterns. Journal of Oncology. 2012;2012:1–10. 10.1155/2012/649498 PMC3345247 22577381

[9] Moolgavkar SH, Venzon DJ. Two-event models for carcinogenesis: incidence curves for childhood and adult tumors. Mathematical Biosciences. 1979;47(1-2):55–77. 10.1016/0025-5564(79)90005-1

[10] Moolgavkar SH, Knudson AG. Mutation and cancer: a model for human carcinogenesis. Journal of the National Cancer Institute. 1981;66(6):1037–52. 6941039 10.1093/jnci/66.6.1037

[11] Luebeck EG, Moolgavkar SH. Multistage carcinogenesis and the incidence of colorectal cancer. Proceedings of the National Academy of Sciences. 2002;99(23):15095–100. 10.1073/pnas.222118199 PMC137549 12415112

[12] Meza R, Jeon J, Moolgavkar SH, Luebeck EG. Age-specific incidence of cancer: Phases, transitions, and biological implications. Proceedings of the National Academy of Sciences. 2008;105(42):16284–9. 10.1073/pnas.0801151105 PMC2570975 18936480

[13] Heidenreich WF, Luebeck EG, Moolgavkar SH. Some properties of the hazard function of the two-mutation clonal expansion model. Risk Analysis. 1997;17(3):391–9. 10.1111/j.1539-6924.1997.tb00878.x 9232020

[14] Meza R, Jeon J, Renehan AG, Luebeck EG. Colorectal cancer incidence trends in the United States and United kingdom: evidence of right- to left-sided biological gradients with implications for screening. Cancer research. 2010;70(13):5419–29. 10.1158/0008-5472.CAN-09-4417 20530677 PMC2914859

[15] Luebeck E, Curtius K, Jeon J, Hazelton W. Impact of tumor progression on cancer incidence curves. Cancer research. 2013;73(3):1086–1096. 10.1158/0008-5472.CAN-12-2198 23054397 PMC3746830

[16] Moolgavkar S, Luebeck G. Two-event model for carcinogenesis: Biological, mathematical, and statistical considerations. Risk Analysis. 1990;10(2):323–341. 10.1111/j.1539-6924.1990.tb01053.x 2195604

[17] Moolgavkar SH, Meza R, Turim J. Pleural and peritoneal mesotheliomas in SEER: age effects and temporal trends, 1973–2005. Cancer Causes & Control. 2009;20(6):935–44. 10.1007/s10552-009-9328-9 19294523

[18] Schöllnberger H, Beerenwinkel N, Hoogenveen R, Vineis P. Cell selection as driving force in lung and colon carcinogenesis. Cancer Research. 2010;70(17):6797–803. 10.1158/0008-5472.CAN-09-4392 20656803 PMC3085130

[19] Little MP, Haylock RGE, Muirhead CR. Modelling lung tumour risk in radon-exposed uranium miners using generalizations of the two-mutation model of Moolgavkar, Venzon and Knudson. International journal of radiation biology. 2002;78(1):49–68. 10.1080/09553000110085797 11747553

[20] Crump KS, Subramaniam RP, Van Landingham CB. A numerical solution to the nonhomogeneous two-stage MVK model of cancer. Risk Analysis. 2005;25(4):921–6. 10.1111/j.1539-6924.2005.00651.x 16268939

[21] Hazelton WD, Moolgavkar SH, Curtis SB, Zielinski JM, Ashmore JP, Krewski D. Biologically based analysis of lung cancer incidence in a large Canadian occupational cohort with low-dose ionizing radiation exposure, and comparison with Japanese atomic bomb survivors. Journal of Toxicology and Environmental Health Part A. 2006;69(11):1013–38. 10.1080/00397910500360202 16840251

[22] Jeon J, Luebeck EG, Moolgavkar SH. Age effects and temporal trends in adenocarcinoma of the esophagus and gastric cardia (United States). Cancer Causes & Control. 2006;17(7):971–81. 10.1007/s10552-006-0037-3 16841264

[23] Jeon J, Meza R, Moolgavkar SH, Luebeck EG. Evaluation of screening strategies for pre-malignant lesions using a biomathematical approach. Mathematical Biosciences. 2008;213(1):56–70. 10.1016/j.mbs.2008.02.006 18374369 PMC2442130

[24] Luebeck EG, Moolgavkar SH, Liu AY, Boynton A, Ulrich CM. Does folic acid supplementation prevent or promote colorectal cancer? Results from model-based predictions. Cancer Epidemiology, Biomarkers & Prevention. 2008;17(6):1360–7. 10.1158/1055-9965.EPI-07-2878 PMC2834311 18539928

[25] Meza R, Luebeck EG, Moolgavkar SH. Gestational mutations and carcinogenesis. Mathematical biosciences. 2005;197(2):188–210. 10.1016/j.mbs.2005.06.003 16087198

[26] Meza R, Jeon J, Moolgavkar S. Quantitative Cancer Risk Assessment of Nongenotoxic Carcinogens In: Hsu CH, Stedeford T, editors. Cancer Risk Assessment. John Wiley & Sons, Inc; 2010 p. 636–658.

[27] Dewanji A, Jeon J, Meza R, Luebeck EG. Number and size distribution of colorectal adenomas under the multistage clonal expansion model of cancer. PLOS Computational Biology. 2011;7(10):e1002213 10.1371/journal.pcbi.1002213 22022253 PMC3192823

[28] Holford TR. The Estimation of Age, Period and Cohort Effects for Vital Rates. Biometrics. 1983;39(2):311–324. 10.2307/2531004 6626659

[29] Holford TR. Understanding the Effects of Age, Period, and Cohort on Incidence and Mortality Rates. Annual Review of Public Health. 1991;12(1):425–457. 10.1146/annurev.pu.12.050191.002233 2049144

[30] Clayton D, Schifflers E. Models for temporal variation in cancer rates. I: Age-period and age-cohort models. Statistics in Medicine. 1987;6(4):449–67. 10.1002/sim.4780060405 3629047

[31] Clayton D, Schifflers E. Models for temporal variation in cancer rates. II: Age-period-cohort models. Statistics in Medicine. 1987;6(4):469–81. 10.1002/sim.4780060405 3629048

[32] Luebeck G, Meza R. Bhat: General likelihood exploration; 2013 R package version 0.9-10. Available from: http://CRAN.R-project.org/package=Bhat.

[33] Gravitt PE, Rositch AF, Silver MI, Marks MA, Chang K, Burke AE, et al A cohort effect of the sexual revolution may be masking an increase in human papillomavirus detection at menopause in the United States. Journal of Infectious Diseases. 2013;207(2):272–80. 10.1093/infdis/jis660 23242540 PMC3532829

[34] Brouwer AF, Eisenberg MC, Carey TE, Meza R. Trends in HPV cervical and seroprevalence and associations between oral and genital infection and serum antibodies in NHANES 2003–2012. BMC Infectious Diseases. 2015;15(1):575 Available from: http://www.biomedcentral.com/1471-2334/15/575. 10.1186/s12879-015-1314-0 26689203 PMC4687319

[35] Kerr WC, Greenfield TK, Bond J, Ye Y, Rehm J. Age, period and cohort influences on beer, wine and spirits consumption trends in the US National Alcohol Surveys. Addiction. 2004;99(9):1111–1120. Available from: http://doi.wiley.com/10.1111/j.1360-0443.2004.00820.x. 10.1111/j.1360-0443.2004.00820.x 15317631

[36] Holford TR, Levy DT, McKay LA, Clarke L, Racine B, Meza R, et al Patterns of birth cohort-specific smoking histories, 1965-2009. American Journal of Preventive Medicine. 2014;46(2):e31–7. Available from: http://www.ncbi.nlm.nih.gov/pubmed/24439359. 10.1016/j.amepre.2013.10.022 24439359 PMC3951759

[37] Meza R, Pourbohloul B, Brunham RC. Birth cohort patterns suggest that infant survival predicts adult mortality rates. Journal of Developmental Origins of Health and Disease. 2010;1(03):174–183. 10.1017/S2040174410000218 25141785

[38] Saba NF, Goodman M, Ward K, Flowers C, Ramalingam S, Owonikoko T, et al Gender and ethnic disparities in incidence and survival of squamous cell carcinoma of the oral tongue, base of tongue, and tonsils: a surveillance, epidemiology and end results program-based analysis. Oncology. 2011;81(1):12–20. 10.1159/000330807 21912193 PMC3186716

[39] Patel SC, Carpenter WR, Tyree S, Couch ME, Weissler M, Hackman T, et al Increasing incidence of oral tongue squamous cell carcinoma in young white women, age 18 to 44 years. Journal of Clinical Oncology. 2011;29(11):1488–94. 10.1200/JCO.2010.31.7883 21383286

