# Supplementary material for: Age Effects and Temporal Trends in HPV-Related and HPV-Unrelated Oral Cancer in the United States: A Multistage Carcinogenesis Modeling Analysis
Source: PLoS One. 2016 Mar 10;11(3):e0151098. doi: 10.1371/journal.pone.0151098 (PMC4786132; doi:10.1371/journal.pone.0151098)
Supplement: S1 File — Provides additional details on subsite classification and model selection, fit, and uncertainty quantification. (PDF) [file pone.0151098.s001.pdf]

Supplemental methods

Subsite classification

Following Chaturvedi et al. (2008) and Brown et al. (2011, 2012), we consider squamous cell carcinomas (SCC) of the head and neck at the following fourteen sites to be HPV-related: C01.9 base of tongue, NOS (not otherwise specified); C02.4 lingual tonsil; C09.0 tonsillar fossa; C09.1 tonsillar pillar; C09.8 overlapping lesion of tonsil; C09.9 tonsil, NOS; C10.0 vallecula; C10.1 anterior surface of epiglottis; C10.2 lateral wall of oropharynx; C10.3 posterior wall of oropharynx; C10.4 branchial cleft; C10.8 overlapping lesion of oropharynx; C10.9 oropharynx, NOS; C14.2 waldeyer ring.

We consider the following twenty-five sites to be HPV-unrelated: C03.0 upper gum; C03.1 lower gum; C03.9 gum, NOS; C04.0 anterior floor of mouth; C04.1 lateral floor of mouth; C04.8 overlapping lesion of floor of mouth; C04.9 floor of mouth, NOS; C05.0 hard palate; C05.1 soft palate, NOS; C05.2 uvula; C05.8 overlapping lesion of palate; C05.9 palate, NOS; C06.0 cheek mucosa; C06.1 vestibule of mouth; C06.2 retromolar area; C06.8 overlapping lesion of other and unspecified mouth; C06.9 mouth, NOS; C12.9 pyriform sinus; C13.0 postcricoid region; C13.1 aryepiglottic fold, hypopharyngeal; C13.2 posterior wall of hypopharynx; C13.8 overlapping lesion of hypopharynx; C13.9 hypopharynx, NOS; C14.0 pharynx, NOS; C14.8 overlapping lesion of lip, oral cavity and pharynx.

We consider the following six oral tongue sites to be HPV-unrelated: C02.0 dorsal surface of tongue, NOS; C02.1 border of tongue; C02.2 ventral surface of tongue, NOS; C02.3 anterior 2/3 of tongue, NOS; C02.8 overlapping lesion of tongue; C02.9 tongue, NOS.

Supplemental results

APC-TSCE model fitting

In Table S1, we present the AIC for the models considered in this analysis. In Figures S1, S2, S3, and S4, we present the incidence rates by period and cohort of oral squamous cell carcinoma among white males, black males, white females, and black females for HPV-related and HPV-unrelated subsite groups as well as the fit of the APC–TSCE model with period and cohort effects.

Two- vs. three-stage model fitting

Because there are significant qualitative differences in the hazards of the two- and three-stage clonal expansion models, we compared fits of three-stage model with APC effects as well. The three-stage model has four degrees of freedom, $\mu_0 X$, μ_1/α, and $p, q = -\frac{1}{2}(\alpha - \beta - \mu_2) \pm \frac{1}{2}\sqrt{(\alpha - \beta - \mu_2)^2 - 4\alpha\mu_2}$. However, because information about $\mu_0 X$ comes only asymptotically (Meza et al., 2008), the model is practically unidentifiable. To this end, we assume $\mu_0 = \mu_1$, and parameterize the model as

$$h_3(t) = r\sqrt{\alpha X}\left(1 - \left(\frac{q - p}{qe^{-pt} - pe^{-qt}}\right)^{r/\sqrt{\alpha X}}\right), \quad (1)$$

where

$$r = \sqrt{(\mu_0 X)(\mu_1/\alpha)}. \tag{2}$$

As parameterized, multiplicative effects on r affect both pre-initiation rates μ_0 and μ_1. We fix $\alpha X = 3 \times 10^7$; the model was insensitive—because of the practical identifiability—to the choice of this combination. In Table S2, we present the AIC for the three-stage models as well.

Uncertainty quantification

Confidence intervals (95%) for the hazards and period and cohort effects are presented in Figure S5 for the APC–TSCE model with period and cohort effects on initiation. Markov chain Monte Carlo (MCMC) methods were used to estimate covariance matrices for the sixteen parameters for each demographic–site pair: using the likelihood as the density function, a multivariate normal posterior distribution was estimated using the Bhat package in R (v3.1). Seeded by the numerical estimates, we used a burn of 1,000 and a run of 10,000.

APC Results

The residual deviance for each unconstrained APC model with natural spline effects is shown Table S3 for each race–gender pair. We use seven degrees of freedom for age, eight for cohort effects, and five for period, corresponding to approximately one degree of freedom for eight, twelve, and eight years respectively. Comparison of values may be made only down columns, not across rows. To avoid issues of identifiability, we use the age–cohort model. The age and cohort effects are plotted in Figure S6

Data	No effects	Period r	Cohort r	Both r	Period p	Cohort p	Both p	Period q	Cohort q	Both q
**HPV-related**										
White men	3231	640	51	0	1309	894	401	1935	1711	1177
Black men	225	169	57	0	84	48	33	86	70	58
White women	222	191	120	0	188	90	58	195	101	90
Black women	86	49	31	0	30	22	9	42	34	33
**HPV-unrelated**										
White men	2272	75	250	0	565	485	157	1095	968	775
Black men	1094	150	195	0	133	256	91	322	361	274
White women	1458	534	49	0	203	137	30	354	211	156
Black women	282	75	23	0	30	34	9	85	78	64
**Oral tongue**										
White men	36	32	13	0	34	11	17	34	10	12
Black men	99	3	13	0	-8	16	-2	11	25	13
White women	102	30	1	0	38	3	6	36	5	11
Black women	2	4	-4	0	5	-6	-2	4	-1	5

Table S1: Akaike Information Criterion (AIC) for APC–TSCE models of incidence of oral squamous cell carcinomas by race and cancer subsite group relative to the model with both period and cohort effects on r. Positive values denote worse relative fits.

Data	No effects	Period r	Cohort r	Both r	Period p	Cohort p	Both p	Period q	Cohort q	Both q
**HPV-related**										
White men	6524	3778	830	857	4163	2297	1901	4701	3620	2165
Black men	663	591	326	145	557	335	264	206	356	173
White women	727	684	561	-9	689	587	422	697	588	252
Black women	231	199	158	-1	193	154	61	198	147	82
**HPV-unrelated**										
White men	2920	754	1674	694	1066	1344	902	1487	1186	1039
Black men	1301	396	654	12	370	551	234	497	480	*
White women	1484	526	347	-3	399	198	73	507	167	111
Black women	344	145	185	14	128	146	66	158	116	119
**Oral tongue**										
White men	225	215	176	0	220	192	49	222	195	200
Black men	135	44	70	-1	39	65	21	53	59	52
White women	121	50	-10	0	48	13	7	42	12	14
Black women	24	27	20	1	28	21	12	27	20	28

Table S2: Akaike Information Criterion (AIC) for APC–3SCE models of incidence of oral squamous cell carcinomas by race and cancer subsite group relative to the APC-TSCE model with both period and cohort effects on r. Positive values denote worse relative fits. *=optimizer did not converge.

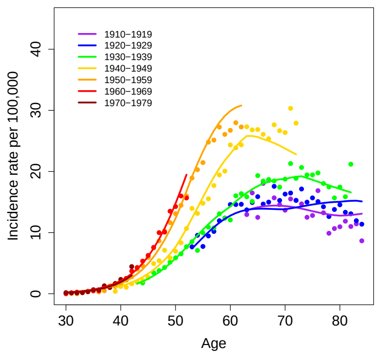

(a) HPV-related cancer incidence by period.

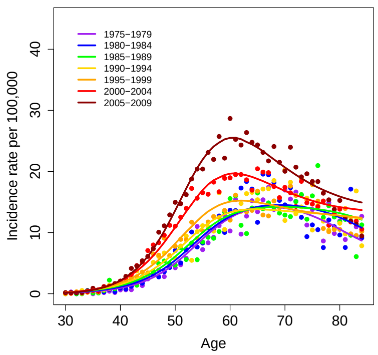

(b) HPV-related cancer incidence by cohort.

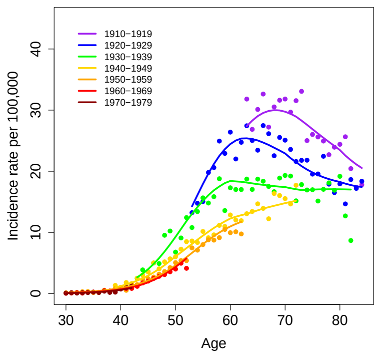

(c) HPV-unrelated cancer incidence by period.

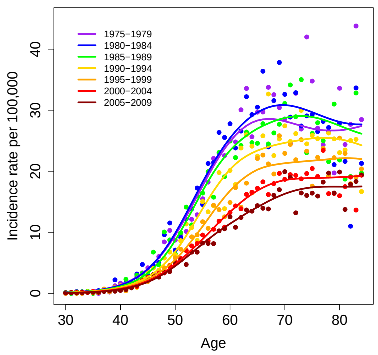

(d) HPV-unrelated cancer incidence by cohort.

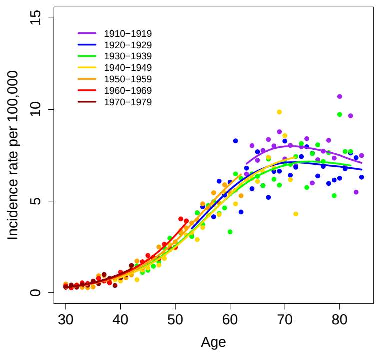

(e) Oral tongue cancer incidence by period.

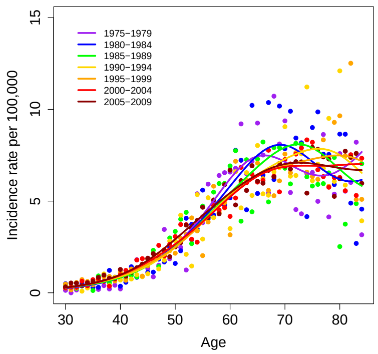

(f) Oral tongue cancer incidence by cohort.

Figure S1: HPV-related, HPV-unrelated, and oral tongue cancer incidence by cohort and period for white men with APC–TSCE model fits. The data are dots and the model fits are lines.

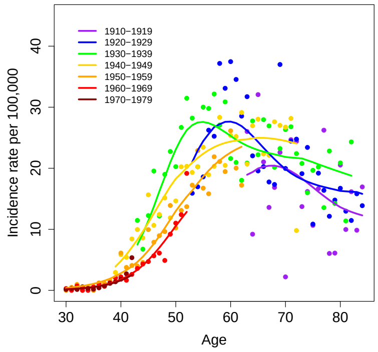

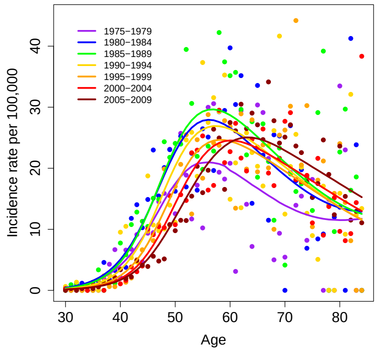

(a) HPV-related cancer incidence by period.

(b) HPV-related cancer incidence by cohort.

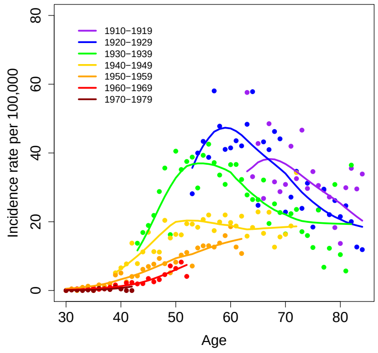

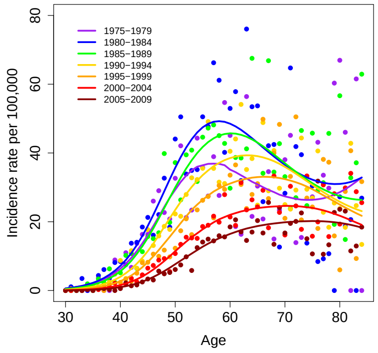

(c) HPV-unrelated cancer incidence by period.

(d) HPV-unrelated cancer incidence by cohort.

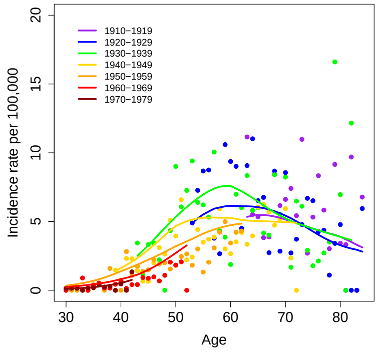

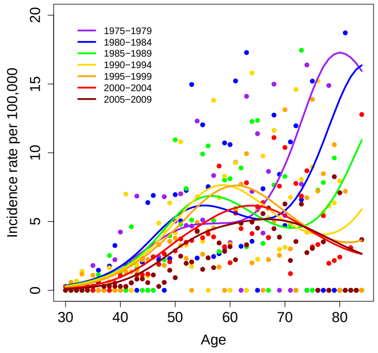

(e) Oral tongue cancer incidence by period.

(f) Oral tongue cancer incidence by cohort.

Figure S2: HPV-related, HPV-unrelated, and oral tongue cancer incidence by cohort and period for black men with APC–TSCE model fits. The data are dots and the model fits are lines.

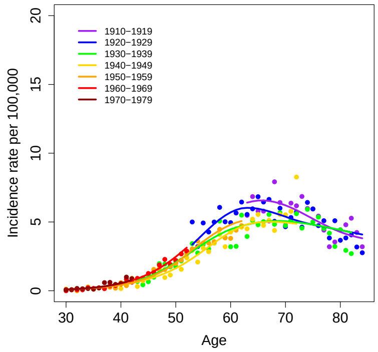

(a) HPV-related cancer incidence by period.

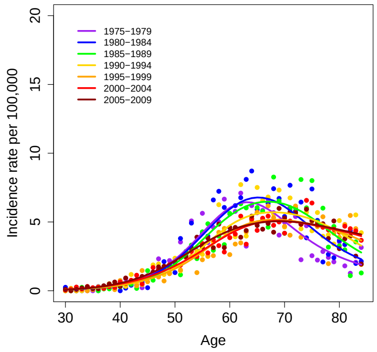

(b) HPV-related cancer incidence by cohort.

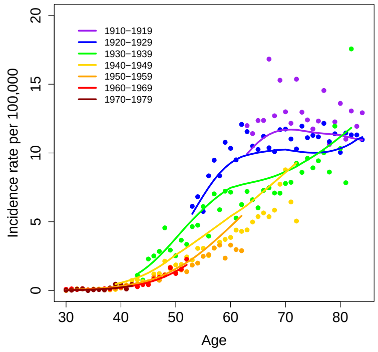

(c) HPV-unrelated cancer incidence by period.

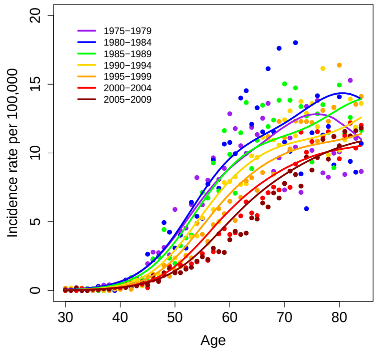

(d) HPV-unrelated cancer incidence by cohort.

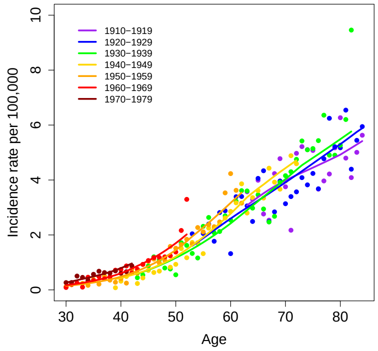

(e) Oral tongue cancer incidence by period.

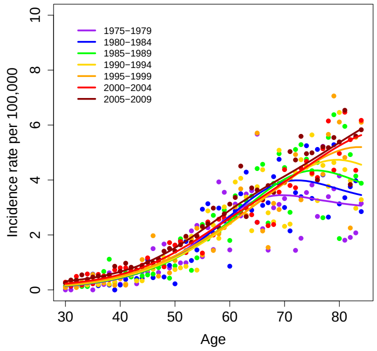

(f) Oral tongue cancer incidence by cohort.

Figure S3: HPV-related, HPV-unrelated, and oral tongue cancer incidence by cohort and period for white women with APC–TSCE model fits. The data are dots and the model fits are lines.

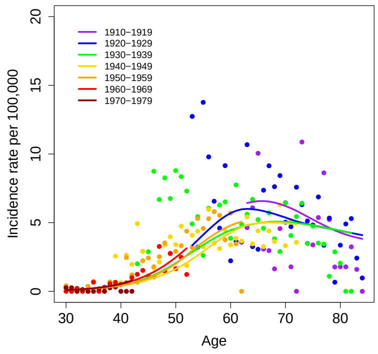

(a) HPV-related cancer incidence by period.

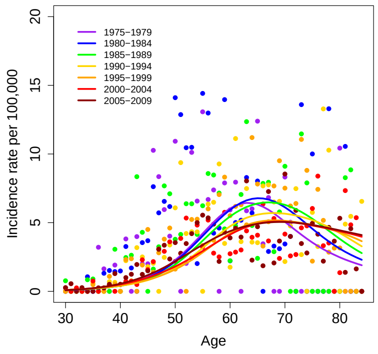

(b) HPV-related cancer incidence by cohort.

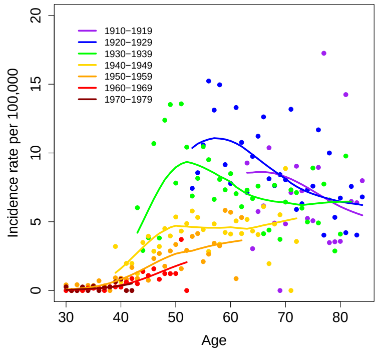

(c) HPV-unrelated cancer incidence by period.

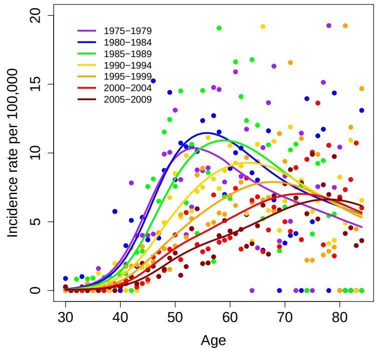

(d) HPV-unrelated cancer incidence by cohort.

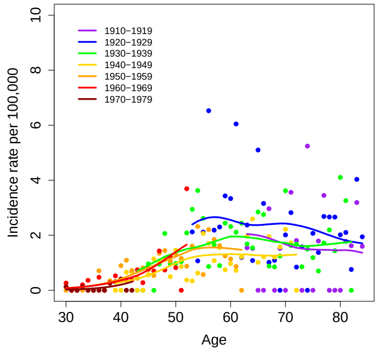

(e) Oral tongue cancer incidence by period.

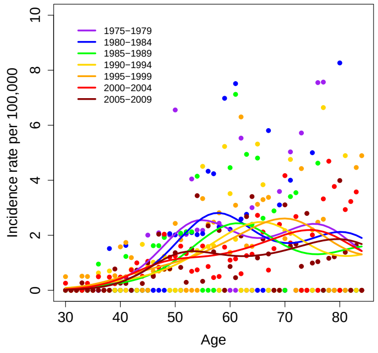

(f) Oral tongue cancer incidence by cohort.

Figure S4: HPV-related, HPV-unrelated, and oral tongue cancer incidence by cohort and period for black women with APC–TSCE model fits. The data are dots and the model fits are lines.

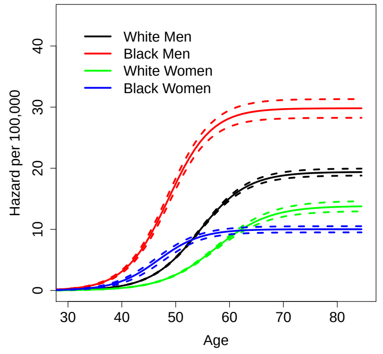

(a) HPV-related hazards.

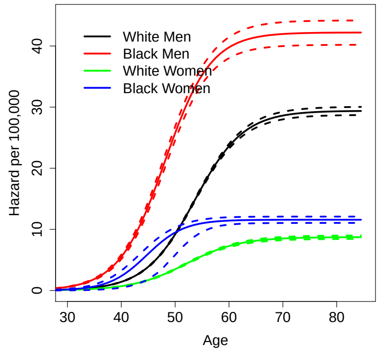

(b) HPV-unrelated hazards.

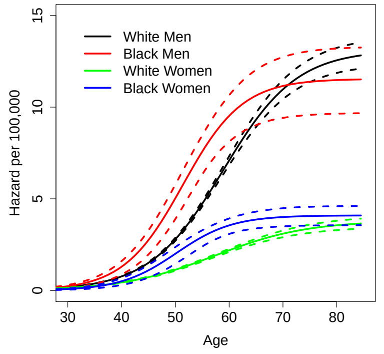

(c) Oral tongue hazards.

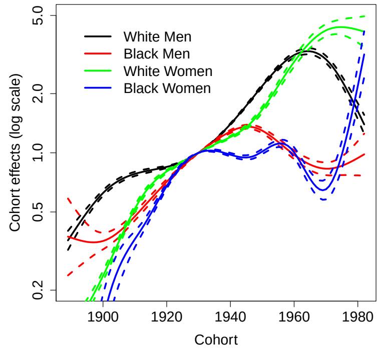

(d) HPV-related cohort effects.

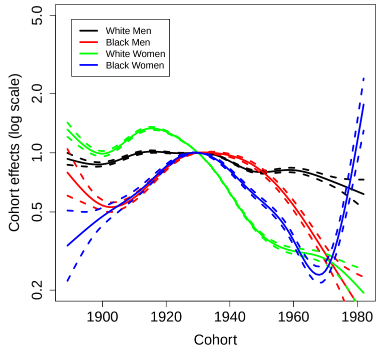

(e) HPV-unrelated cohort effects.

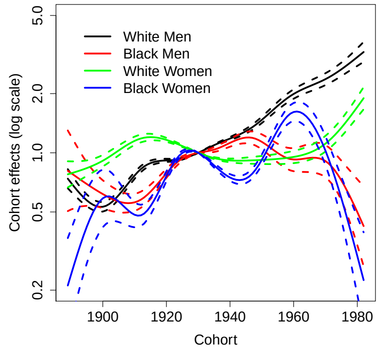

(f) Oral tongue cohort effects.

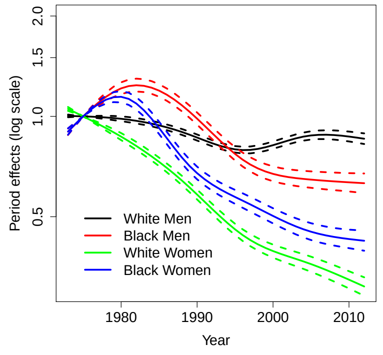

(g) HPV-related period effects.

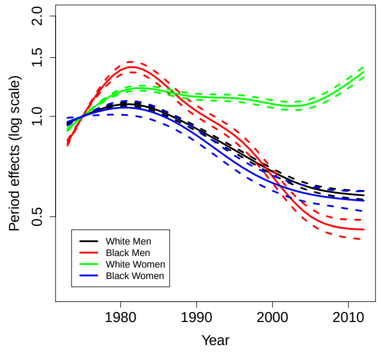

(h) HPV-unrelated period effects.

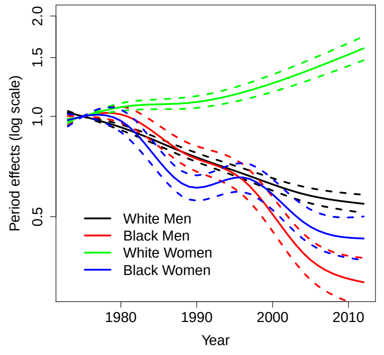

(i) Oral tongue period effects.

Figure S5: Hazard, cohort effects, and period effects for the cohort-and-period-effects-on-r APC–TSCE models of oral squamous cell carcinoma by race and cancer subsite group with 95% confidence intervals.

Model	White men Residual Dev.	Black men Residual Dev.	White women Residual Dev.	Black women Residual Dev.
**HPV-related**				
Age–Cohort	2380.7	2262.1	2193.3	1869.5
Age–Period–Cohort	2325.9	2229.0	2165.7	1853.0
Age–Period	2649.2	2346.3	2272.9	1896.7
**HPV-unrelated**				
Age–Cohort	2462.7	2277.8	2266.2	2088.7
Age–Period–Cohort	2358.5	2187.7	2202.4	2081.6
Age–Period	2425.6	2339.8	2674.4	2166.9
**Oral tongue**				
Age–Cohort	2504.8	1850.1	2367.9	1324.2
Age–Period–Cohort	2495.4	1837.3	2352.7	1316.5
Age–Period	2518.1	1850.0	2393.4	1334.8

Table S3: Residual deviance for APC model fits of oral squamous cell carcinoma incidence by race and cancer subsite group.

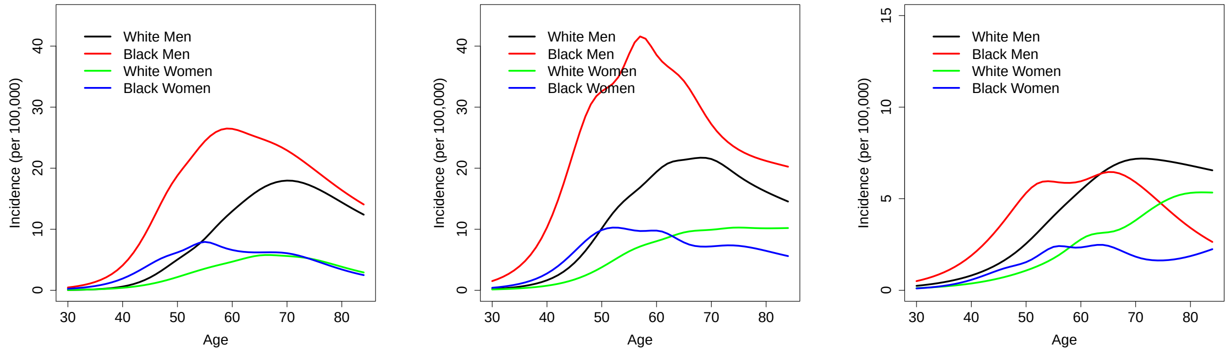

(a) HPV-related age effects. (b) HPV-unrelated age effects. (c) Oral tongue age effects.

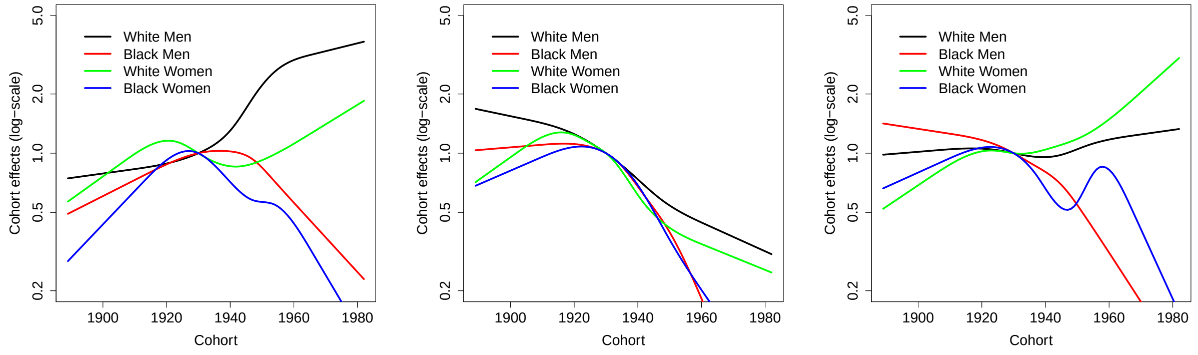

(d) HPV-related cohort effects. (e) HPV-unrelated cohort effects. (f) Oral tongue cohort effects.

Figure S6: Age and cohort effects for APC models of oral squamous cell carcinoma incidence by race and cancer subsite group.
